# Association between Autoimmune Rheumatic Diseases and the Risk of Dementia

**DOI:** 10.1155/2014/861812

**Published:** 2014-04-30

**Authors:** Kang Lu, Hao-Kuang Wang, Chih-Ching Yeh, Chih-Yuan Huang, Pi-Shan Sung, Liang-Chao Wang, Chih-Hsin Muo, Fung-Chang Sung, Han-Jung Chen, Ying-Chun Li, Li-Ching Chang, Kuen-Jer Tsai

**Affiliations:** ^1^Department of Neurosurgery, E-Da Hospital, I-Shou University, Kaohsiung 824, Taiwan; ^2^Institute of Health Care Management, National Sun Yat-Sen University, Kaohsiung 804, Taiwan; ^3^Institute of Clinical Medicine, National Cheng Kung University, Tainan 704, Taiwan; ^4^School of Public Health, College of Public Health and Nutrition, Taipei Medical University, Taipei 110, Taiwan; ^5^Department of Public Health, China Medical University, Taichung, Taiwan; ^6^Neurosurgical Service, Department of Surgery, National Cheng Kung University Hospital, Tainan 704, Taiwan; ^7^Department of Neurology, National Cheng Kung University Hospital, Tainan 704, Taiwan; ^8^Management Office for Health Data, China Medical University Hospital, Taichung 404, Taiwan; ^9^Department of Occupational Therapy, I-Shou University, Kaohsiung 824, Taiwan; ^10^Center of Clinical Medicine, National Cheng Kung University Hospital, College of Medicine, National Cheng Kung University, Tainan 704, Taiwan

## Abstract

*Aim*. Autoimmune rheumatic diseases (ARD) are characterized by systemic inflammation and may affect multiple organs and cause vascular events such as ischemic stroke and acute myocardial infarction. However, the association between ARD and increased risk of dementia is uncertain. This is a retrospective cohort study to investigate and compare the risk of dementia between patients clinically diagnosed with ARD and non-ARD patients during a 5-year follow-up period. *Methods*. Data were obtained from the Longitudinal Health Insurance Database 2000 (LHID2000). We included 1221 patients receiving ambulatory or hospitalization care and 6105 non-ARD patients; patients were matched by sex, age, and the year of index use of health care. Each patient was studied for 5 years to identify the subsequent manifestation of dementia. The data obtained were analyzed by Cox proportional hazard regression. *Results*. During the 5-year follow-up period, 30 ARD (2.48%) and 141 non-ARD patients (2.31%) developed dementia. During the 5-year follow-up period, there were no significant differences in the risks of any type of dementia (adjusted hazard ratio (HR), 1.18; 95% CI, 0.79–1.76) in the ARD group after adjusting for demographics and comorbidities. *Conclusions*. Within the 5-year period, patients with and without ARD were found to have similar risks of developing dementia.

## 1. Introduction

Autoimmune rheumatic diseases (ARD) such as rheumatoid arthritis (RA), systemic lupus erythematosus (SLE), Sjögren syndrome, progressive systemic sclerosis (PSS), polymyositis (PM), dermatomyositis (DM), vasculitides (including polyarteritis nodosa, Kawasaki disease, hypersensitivity angiitis, Wegener's granulomatosis, giant cell arteritis, and Takayasu arteritis), and Behçet's disease are chronic diseases that are characterized by progressive and systemic inflammation.

Patients with ARD have a higher mortality rate and a greater risk of developing cancer than the general population [1–5]. ARD may affect multiple organs and cause vascular events such as ischemic stroke, acute myocardial infarction, and peripheral arterial occlusive disease [6–10]. Multiple epidemiological studies have shown that ARD is a risk factor for dementia and other neurological conditions such as stroke and seizure [6, 9, 11]. However, some studies have reported contrary results that ARD had a lower prevalence of dementia and decreased risk of AD [12–15]. Studies that reported ARD as a significant risk factor for dementia exhibited small case series or lacked a definitive diagnosis for dementia [16, 17]. Therefore, the association between ARD and dementia has not been fully established thus far [12–18].

This study aimed to determine the association between ARD and increased risk and incidence of dementia using the Longitudinal Health Insurance Database (LHID2000) in Taiwan and to determine if such an association could be explained based on medical comorbidities or ARD.

## 2. Methods

### 2.1. Database, Standard Protocol Approval, Registration, and Patient Consent

This study used data from the LHID2000, which is a subset of the National Health Insurance Research Database (NHIRD) in Taiwan. Taiwan began its National Health Insurance (NHI) program on March 1, 1995, in order to provide comprehensive health care to all citizens of Taiwan. As of December 2007, 22.6 million individuals, representing 98% of the Taiwanese population, had been enrolled in the program [8, 19]. The data on NHI claims include comprehensive demographic data, dates of clinical visits, and diagnostic codes, as well as details of prescriptions, examinations, and procedures, which are entered into the NHIRD; it is maintained by the Bureau of National Health Insurance, Taiwan. Scientists can access the data for research purposes. The LHID2000 consists of information on the original claims and registration files of 1,000,000 individuals, randomly sampled from the NHIRD in 2000. Taiwan National Health Research Institutes (TNHRI) (http://nhird.nhri.org.tw/) publishes the information on the creation of the database online. There are no significant differences in gender, age, or the amount of insurance payments between the individuals registered in LHID2000 and the individuals in the NHI program. More than 600 studies have been published using the data obtained from the LHID2000. The Institutional Review Board exempted this study from a full review.

This is a retrospective cohort study. Patients with ARD were identified from the catastrophic illness registry in the LHID2000. In Taiwan, ARD patients are eligible for a catastrophic illness certificate after a rheumatology specialist makes the diagnosis based on clinical manifestations, laboratory data, and the criteria set by the American College of Rheumatology, which is reviewed by rheumatologists commissioned by the NHI. Individuals with these certificates are eligible for government subsidies, which include discounted outpatient and inpatient copayments [1–8]. Thus, the catastrophic illness patient data is highly accurate and reliable.

We identified patients with an ARD diagnosis using the International Classification of Diseases, ninth revision, Clinical Modification (ICD-9-CM) code (714.0 for rheumatoid arthritis (RA), 710.0 for systemic lupus erythematosus (SLE), 710.2 for Sjögren syndrome, 710.1 for progressive systemic sclerosis (PSS), 710.3 for polymyositis (PM), 710.4 for dermatomyositis (DM), vasculitides (446.0 for polyarteritis nodosa, 446.1 for Kawasaki disease, 446.2 for hypersensitivity angiitis, 446.4 for Wegener's granulomatosis, 446.5 for giant cell arteritis, and 446.7 for Takayasu disease), and 136.1 for Behçet's diseases) from the registry of catastrophic illnesses from 2000 to 2005. Patients found to have ARD prior to 2000 were excluded from the study. Additionally, patients diagnosed with the following conditions prior to the index use of health care facilities were excluded from the study: 290.0 (senile dementia, uncomplicated); 290.1 (presenile dementia); 290.2 (senile dementia with delusional or depressive features); 290.3 (senile dementia with delirium); 290.4 (arteriosclerotic dementia); 294.1 (dementia in conditions classified elsewhere); 331.0 (Alzheimer's disease, AD); 331.1 (pick disease); and 331.2 (senile degeneration of brain) [20]. A total of 1221 patients with new diagnoses of ARD from 2000 to 2005 were selected for the study.

Subjects for the comparison cohort were selected from the remaining records of patients in the LHID2000 registry. First, we excluded patients with previous NHI ambulatory claim records or inpatient records indicating any ARD diagnosis between 1996 and 1999. Then, we randomly selected 6105 beneficiaries (5 comparison subjects were randomly selected for every patient with ARD) matched in terms of sex, age (<30, 30–49, and ≥50 years), and the year of index use of health care services. We defined the first ambulatory care visit occurring in the year of index health care use as their index health care use. Patients who were diagnosed with incident dementia prior to their clinic visits were excluded from the comparison cohort group. There were a total of 7326 patients enrolled in the study. In order to identify patients who subsequently suffered from dementia (ICD-9-CM codes from 290.0 to 290.4, 294.1, and from 331.0 to 331.2), every patient was followed up until 2010 using data on their index health care visit.

### 2.2. Statistical Analysis

The primary aim of this study was to identify patients who had received ambulatory care or undergone hospitalization for any type of dementia. We used Pearson's *χ* ^2^ tests to compare differences between patients with and without ARD at baseline in terms of demographic characteristics (age, sex, and geographical location of the patient's residence (Northern, Central, Eastern, and Southern Taiwan)) and certain comorbidities (diabetes, hyperlipidemia, hypertension, cerebrovascular disorders, heart failure (HF), and atrial fibrillation (AF)) [21–23]. These comorbidities were considered only if the condition occurred in an inpatient setting or during 2 or more ambulatory care claims within 1 year, prior to the index ambulatory care visit.

Patients without ARD were designated as the reference group. The crude and adjusted hazard ratios (HR) for the association between ARD and dementia during the 5-year follow-up period were estimated using univariate and multivariate Cox proportion hazard regression. The demographic characteristics and selected comorbidities were corrected for determine the adjusted HR. All models were examined for violation of the proportional hazards assumption and the results showed no violations of the proportionality assumption. A stratified Cox proportional hazard regression was performed to compare the relationship between ARD and dementia in various ARD groups. The associations of ARD with dementia were further analyzed according to dementia subtypes. The dementia subtypes were as follows: AD (ICD-9-CM code 331.0), vascular dementia (ICD-9-CM code 290.4), and unspecified dementia (ICD-9-CM codes 290.0–290.3, 294.1, and 331.1-331.2). The HR values and 95% confidence intervals (CI) were computed with a statistical significance of 0.05. All statistical analyses were conducted using the SAS statistical package (SAS System for Windows, version 9.1, SAS Institute Inc., Cary, NC, USA).

## 3. Results

We identified 1221 ARD and 6105 non-ARD cases in our analysis. The data description for the sampled subjects is presented in Table 1. There were no significant difference in age or sex between the ARD and non-ARD patients, after matching for gender, age in the 5-year interval, and year of index health care use. However, among subjects with ARD, the female-to-male ratio and age distribution varied according to the ARD subtype. The prevalence of ARD was higher in female subjects (79.4%). In decreasing order, the female-to-male ratios were as follows: SLE (87.3%), Sjögren syndrome (86.7%), RA (75.8%), PSS (74.3%), PM/DM (71.9%), Behçet's disease (60.0%), and Vasculitides (55.6%). Most patients with RA, SS, and PM/DM were older than 50 years. Patients with PSS and Behçet's disease were aged 30–49, and SLE patients were aged less than 30. ARD patients had a higher prevalence of HF (8.93 versus 5.44%, *P* < 0.001) and AF (0.90 versus 0.62%, *P* = 0.028) than patients without ARD, after matching for age and sex.

The crude and adjusted HRs for dementia during the 5-year follow-up period after index health care use are shown in Table 2. Out of 7326 patients, 171 patients experienced dementia during the 5-year follow-up period, including 30 patients (2.48% of the patients with ARD) from the study cohort and 141 patients (2.31% of the patients without ARD) from the comparison cohort. The univariate Cox proportional hazard regressions showed that the HR for dementia in ARD patients within the 5-year period was 1.10 (95% CI, 0.74–1.63; *P* = 0.63) compared to that in non-ARD patients. We further analyzed the HR value after adjusting the data for diabetes, hyperlipidemia, hypertension, cerebrovascular disorders, heart failure, and atrial fibrillation. The HR value of 1.18 (95% CI, 0.79–1.76; *P* = 0.41) was obtained. The HRs for dementia in both cohorts with the ARD subtypes RA, SLE, and SS were 1.42, 0.71, and 1.31, respectively. The adjusted HRs for RA, SLE, and SS were 1.20, 2.86, and 0.81, respectively. Table 2 also presents HR of dementia between cohorts according to different age groups. Stratified Cox proportional hazard regressions yielded the following results for the 5-year period: in the age group of <30 years, the HR of patients with ARD was 1.29 (95% CI, 0.81–1.57; *P* = 0.56), in comparison with the non-ARD patients; in the age group of 30–49 years, the HR of ARD patients was 1.31 (95% CI, 0.67–1.63; *P* = 0.34). The HR for TBI patients older than 50 years was 1.08 (95% CI, 0.76–1.52; *P* = 0.59). Furthermore, we found that the adjusted HR for dementia within the 5-year period in ARD patients younger than 30 years was 1.23 (95% CI, 0.76–1.61; *P* = 0.62); the corresponding value in ARD patients aged 30–49 was 1.29 (95% CI, 0.73–1.59; *P* = 0.62); and in patients older than 50 years, the HR values for TBI patients were 1.21 (95% CI, 0.81–1.77; *P* = 0.44).

Table 3 shows the HR analysis for dementia subtypes in both cohorts. The HRs for vascular dementia in both cohorts for ARD and RA were 0.86 and 1.66, respectively. The adjusted HRs for ARD and RA were 0.99 and 1.52, respectively. The HRs for unspecific dementia in both cohorts for ARD, RA, SLE, and SS were 1.21, 1.51, 0.84, and 1.54, respectively. The adjusted HRs for ARD, RA, SLE, and SS were 1.28, 1.27, 3.48, and 0.91, respectively.

## 4. Discussion

There has been interest in the potential association between ARD and neuropsychiatric affections, including cognitive dysfunction, headache, mood disorder, cerebrovascular changes within intracranial vessels, seizures, polyneuropathy, anxiety, and psychosis [24, 25]. The primary findings of our study showed that the ARD and comparison groups had similar risks of developing dementia during the 5-year follow-up. We observed that 2.48% of ARD patients experienced dementia (30 patients), and 2.31% of non-ARD patients (141 patients) developed dementia. However, there were no significant differences in the risks of dementia in the ARD group after adjusting for demographics and comorbidities.

The mechanism leading to a high incidence of dementia in patients with ARD is not fully understood. There are several reasons why ARD patients may have an increased risk of developing dementia. Patients with ARD have a well-documented risk for subclinical and clinical cardiovascular diseases such as stroke and myocardial infraction [6–9]. Epidemiological studies have shown that the risk of dementia increases with cardiovascular abnormalities such as coronary heart disease, heart failure, and atrial fibrillation. Thus, ARD can increase the incidence of dementia [16, 17]. In addition, ARD is a chronic inflammatory disease, which is characterized by progressive and systemic inflammation. Such inflammation is associated with an increased risk for cognitive decline due to a dysfunction in the hypothalamic-pituitary-adrenal axis (HPA) [26–28].

Our results, however, did not support the theory that ARD is a potential risk factor for dementia. Trysberg et al. showed that the low levels of amyloid *β* found in SLE patients seemed to be a direct consequence of diminished production of amyloid precursor protein, probably mediated by heavy anti-inflammatory/immunosuppressive therapy [29–31]. In Taiwan, most ARD patients who had a catastrophic illness certificate were treated with anti-inflammatory drugs for musculoskeletal symptoms unless there were specific contraindications. To further investigate the effect of anti-inflammatory drugs, we should have compared the risk of developing dementia in patients using anti-inflammatory/immunosuppressive therapy with that of patients who are not using these drugs. This study was unable to make such a comparison, as it did not have a prospective randomized control design. Additionally, in Taiwan, it is unacceptable to stop the treatment of ARD patients with catastrophic illness certificate. Therefore, the ARD group with anti-inflammatory/immunosuppressive therapy is less likely to develop dementia during the 5-year follow-up when compared to the control group.

After adjusting for sociodemographic characteristics, region of residence, and selected comorbidities, SLE diagnosis was independently associated with an adjusted HR of 2.86 and 3.48, respectively. Most SLE patients were aged less than 30 in our study. Young onset dementia more likely has a genetic or metabolic disease. These findings suggest that patients with SLE seem to have higher cognitive dysfunction after long-term follow-up.

Our study had several limitations. First, the sample size was not large enough. A huge sample size is necessary to detect differences in dementia for patients with or without ARD, considering the overall low incidence of dementia and the lower incidence attributed to ARD. Therefore, the difference in incidence rate between ARD and comparison groups is low; the statistical power is too low to detect the difference. Second, the NHI database included data on patients that had treatment for ARD and dementia, but it did not indicate parameters such as clinical severity and laboratory data (serum cholesterol levels, titers of antiphospholipid antibodies, or rheumatic factor). Marital status, body mass index, smoking habits, intelligence, and education level are also associated with ARD and dementia [10–12]. However, data on these variables were not available and could not be adjusted for in the analysis. Third, dementia is more likely to occur in the elderly; therefore, selecting younger patients may affect the age-related risks. All ARDs were included in the present study. Thus, patients with different forms of ARDs had varying ages. Both RA and SS cohorts consisted of older patients. The future analysis of each type of ARD may yield results that are more concise. However, age is not a risk factor for dementia after performing a stratified subgroup analysis. Forth, patients with ARD have an increased risk for cancers and greater mortality than the general population [1–4]. If there were more numbers of deaths among patients with ARD, it would cause an underestimation of the association and increase the risk of dementia. Fifth, ARD diagnosis was based on ICD-9-CM database. The duration or subtypes of ARD were hard to know. For example, Sjögren's syndrome is classified into primary and secondary diseases. Primary Sjögren's syndrome presents alone in the absence of any other autoimmune disorders and only impacts the salivary and lacrimal glands. Secondary disease refers to Sjögren's syndrome in individuals who have another autoimmune disease, such as RA or SLE. It is difficult to know primary or secondary Sjögren's syndrome within the ICD-9-CM-derived database. Lastly, AD is not the most common dementia subtype in our study. The most common forms of dementia are AD, vascular dementia, frontotemporal dementia, semantic dementia, and dementia with Lewy bodies. However, patients with dementia can apply the catastrophic illness registry only when patients with a dementia diagnosis using the ICD-9-CM code 290 in Taiwan. This situation causes vascular dementia or Alzheimer's disease underestimation. Therefore, it is hard to know what subtype of dementia is related to ARD in our study.

## 5. Conclusions

Our study suggests that patients with ARD have similar chances of developing dementia during the 5-year follow-up compared to the control group. Although evidence suggests that ARD is not a risk factor for dementia, a lot of limitations preclude formal conclusions. Care must be taken before extrapolating these results further due to some limitation, such as the low difference in incidence rate between ARD and comparison groups. Therefore, further research is needed to address this issue.

## Figures and Tables

**Table 1:** Comparison of demographic characteristics and comorbidities of ARD and non-ARD patients.

Variable	ARD *N* = 1221		Comparison *N* = 6105		*P*
	*n*	%	*n*	%	
Sex					1
Male	251	20.6	1255	20.6
Female	970	79.4	4850	79.4
Age, year					1
<30	228	18.7	1140	18.7
30–49	433	35.5	2165	35.5
≥50	560	45.9	2800	45.9
Geographic region					0.003
Northern	507	41.5	2900	47.5
Central	299	24.5	1200	19.7
Southern	336	27.5	1713	28.1
Eastern	79	6.47	292	4.78
Comorbidities					
Diabetes	116	9.50	538	8.81	0.294
Hyperlipidemia	187	15.3	896	14.7	0.655
Hypertension	209	17.1	1054	17.3	0.814
Stroke	28	2.29	119	1.95	0.623
Heart failure	109	8.93	332	5.44	<0.001
Atrial fibrillation	11	0.90	38	0.62	0.028

**Table 2:** Dementia risk among sampled patients of different age groups during the 5-year follow-up period from index health care utilization (*N* = 7326).

Dementia	Comparison	All	RA	SLE	SS
	*N* = 6105	*N* = 1221	*N* = 637	*N* = 300	*N* = 173
Total (%)	141 (2.31)	30 (2.48)	20 (3.14)	5 (1.67)	5 (2.89)
Crude HR (95% CI)	1.00	1.10 (0.74–1.63)	1.42 (0.89–2.27)	0.71 (0.29–1.74)	1.31 (0.54–3.20)
*P* value		0.63	0.24	0.46	0.46
Adjusted HR (95% CI)	1.00	1.18 (0.79–1.76)	1.20 (0.75–1.92)	2.86 (1.16–7.05)	0.81 (0.33–1.99)
*P* value		0.41	0.56	0.02	0.64

Age group					
<30	7 (0.11)	2 (0.16)	1 (0.16)	1 (0.33)	0 (0.00)
Crude HR (95% CI)	1.00	1.29 (0.81–1.57)	1.22 (0.77–1.96)	2.73 (0.68–10.99)	—
*P* value		0.56	0.36	0.67	
Adjusted HR (95% CI)	1.00	1.23 (0.76–1.61)	1.12 (0.82–2.04)	3.83 (0.89–16.55)	—
*P* value		0.62	0.53	0.45	
30–49	16 (0.26)	4 (0.33)	3 (0.47)	1 (0.33)	0 (0.00)
Crude HR (95% CI)	1.00	1.31 (0.67–1.63)	1.91 (0.86–2.41)	1.62 (0.71–2.32)	—
*P* value		0.34	0.48	0.67	
Adjusted HR (95% CI)	1.00	1.29 (0.73–1.59)	1.87 (0.81–2.39)	1.48 (0.88–2.45)	—
*P* value		0.62	0.53	0.45	
≥50	118 (1.82)	24 (1.80)	16 (2.51)	3 (1.00)	5 (2.89)
Crude HR (95% CI)	1.00	1.08 (0.76–1.52)	1.38 (0.91–2.33)	0.79 (0.34–1.87)	1.52 (0.57–3.75)
*P* value		0.59	0.27	0.61	0.43
Adjusted HR (95% CI)	1.00	1.21 (0.81–1.77)	1.24 (0.82–1.89)	0.800 (0.34–1.90)	1.79 (0.51–2.03)
*P* value		0.44	0.47	0.53	0.47

Hazard ratio was calculated by using Cox proportional regression method during the 5-year follow-up period. Adjustments were made for demographic characteristics (age, sex, and the geographical region) and selected comorbidities in patients (diabetes, hyperlipidemia, hypertension, stroke, heart failure, and atrial fibrillation).

**Table 3:** Crude and adjusted hazard ratios by dementia subtype among sampled patients during the 5-year follow-up from index health care utilization.

Dementia	Comparison	All	RA	SLE	SS
	*N* = 6105	*N* = 1221	*N* = 637	*N* = 300	*N* = 173
Alzheimer's disease					
Yes (%)	9 (0.15)	0 (0.00)	0 (0.00)	0 (0.00)	0 (0.00)
Crude HR (95% CI)	1.00				
Adjusted HR (95% CI)	1.00				
Vascular dementia					
Yes (%)	12 (0.20)	2 (0.16)	2 (0.31)	0 (0.00)	0 (0.00)
Crude HR (95% CI)	1.00	0.86 (0.19–3.82)	1.66 (0.37–7.42)	—	—
*P* value		0.43	0.51		
Adjusted HR (95% CI)	1.00	0.99 (0.22–4.45)	1.52 (0.34–6.86)	—	—
*P* value		0.51	0.37		
Unspecific dementia					
Yes (%)	120 (1.97)	28 (2.29)	18 (2.83)	5 (1.67)	5 (2.89)
Crude HR (95% CI)	1.00	1.21 (0.80–1.82)	1.51 (0.92–2.47)	0.84 (0.34–2.05)	1.54 (0.63–3.78)
*P* value		0.57	0.33	0.68	0.41
Adjusted HR (95% CI)	1.00	1.28 (0.84–1.93)	1.27 (0.77–2.10)	3.48 (1.40–8.63)	0.91 (0.37–2.26)
*P* value		0.32	0.24	0.03	0.46

Hazard ratio was calculated using Cox proportional regression method during the 5-year follow-up period. Adjustments were made for demographic characteristics (age, sex, and the geographical region) and selected comorbidities in the patients (diabetes, hyperlipidemia, hypertension, stroke, heart failure, and atrial fibrillation).

## Abbreviations

ARD: Autoimmune rheumatic diseases
DM: Dermatomyositis
HR: Hazard ratio
ICD-9-CM: International Classification of Diseases, Ninth Revision Clinical Modification
HPA: Hypothalamic-pituitary-adrenal axis
LHID2000: Longitudinal Health Insurance Database 2000
NHI: National Health Insurance
NHIRD: National Health Insurance Research Database
PM: Polymyositis
PSS: Progressive systemic sclerosis
RA: Rheumatoid arthritis
SLE: Systemic lupus erythematosus
TNHRI: Taiwan National Health Research Institutes.
